# Adiposity and Blood Pressure in 110 000 Mexican Adults

**DOI:** 10.1161/HYPERTENSIONAHA.116.08791

**Published:** 2017-03-08

**Authors:** Louisa Gnatiuc, Jesus Alegre-Díaz, Jim Halsey, William G. Herrington, Malaquías López-Cervantes, Sarah Lewington, Rory Collins, Roberto Tapia-Conyer, Richard Peto, Jonathan R. Emberson, Pablo Kuri-Morales

**Affiliations:** From the Clinical Trial Service Unit and Epidemiological Studies Unit (L.G., J.H., W.G.H., S.L., R.C., R.P., J.R.E.) and Medical Research Council Population Health Research Unit (S.L., J.R.E.), Nuffield Department of Population Health, University of Oxford, United Kingdom; and School of Medicine, National Autonomous University of Mexico (Universidad Nacional Autónoma de México) (J.A.-D., M.L.-C., R.T.-C., P.K.-M.).

**Keywords:** adiposity, blood pressure, cross-sectional studies, Mexico, obesity

## Abstract

Previous studies have reached differing conclusions about the importance of general versus central markers of adiposity to blood pressure, leading to suggestions that population-specific adiposity thresholds may be needed. We examined the relevance of adiposity to blood pressure among 111 911 men and women who, when recruited into the Mexico City Prospective Study, were aged 35 to 89 years, had no chronic disease, and were not taking antihypertensives. Linear regression was used to estimate the effects on systolic and diastolic blood pressure of 2 markers of general adiposity (body mass index and height-adjusted weight) and 4 markers of central adiposity (waist circumference, hip circumference, waist:hip ratio, and waist:height ratio), adjusted for relevant confounders. Mean (SD) adiposity levels were: body mass index (28.7±4.5 kg/m^2^), height-adjusted weight (70.2±11.2 kg), waist circumference (93.3±10.6 cm), hip circumference (104.0±9.0 cm), waist:hip ratio (0.90±0.06), and waist:height ratio (0.60±0.07). Associations with blood pressure were linear with no threshold levels below which lower general or central adiposity was not associated with lower blood pressure. On average, each 1 SD higher measured adiposity marker was associated with a 3 mm Hg higher systolic blood pressure and 2 mm Hg higher diastolic blood pressure (SEs <0.1 mm Hg), but for the waist:hip ratio, associations were only approximately half as strong. General adiposity associations were independent of central adiposity, but central adiposity associations were substantially reduced by adjustment for general adiposity. Findings were similar for men and women. In Mexican adults, often overweight or obese, markers of general adiposity were stronger independent predictors of blood pressure than measured markers of central adiposity, with no threshold effects.

## Abstract 2

Supplemental Digital Content is available in the text.

Adiposity and blood pressure are major modifiable risk factors for cardiovascular and other chronic diseases.^1–4^ Levels of adiposity^5^ and blood pressure^6^ are increasing in many populations and are estimated to be responsible for a substantial proportion of morbidity and mortality globally.^7^ Body mass index (BMI), the most commonly used marker of adiposity, is strongly related to blood pressure,^1^ and Mendelian randomization studies^8–10^ and randomized intervention trials of weight-loss^11,12^ suggest this relationship to be causal. A recent study of 0.5 million mostly lean Chinese adults found that systolic blood pressure (SBP) increased by ≈8 mm Hg for each 5 kg/m^2^ higher BMI,^13^ somewhat greater than has been documented in Western populations.^1^ In addition to varying between populations, the relevance of adiposity to blood pressure may vary within a population over time, perhaps because of changing levels of other characteristics. Different markers of adiposity also differ in their relevance to blood pressure. For example, although some studies have found BMI to be more strongly related to blood pressure than waist circumference (WC) or the waist:hip ratio (WHR),^13,14^ others have found the opposite,^15–17^ whereas BMI and the waist:height ratio (WHtR) have been found to exhibit similar associations with blood pressure in some studies.^14,18^

In 2008, a World Health Organization Expert Consultation on WC and WHR^19^ recommended that further studies are needed to determine whether existing recommended adiposity cutoff points should be population specific and noted that there was little existing evidence among Hispanic populations. We report the relevance of several common markers of adiposity to blood pressure among 110 000 men and women from Mexico City who were free from disease and not taking antihypertensives when recruited into a prospective study at the start of the 21st century.^20,21^

## Methods

The rationale and design of the Mexico City Prospective Study has been described in detail previously^20,21^; a summary is given below.

### Recruitment and Baseline Assessment

Between 1998 and 2004, 159 755 participants aged ≥35 years from the Coyoacán and Iztapalapa districts of Mexico City were visited in their homes and agreed to take part in a prospective cohort study. Trained nurses recorded data directly into a handheld electronic device, with subsequent automated checks for unusual values or data entry errors. Age, sex, socioeconomic status, lifestyle (tobacco use, alcohol consumption, physical activity, and diet), disease history, and medication were recorded.^20^ Weight (wearing light clothes), standing height, WC, and hip circumference (HC) were measured to the nearest 0.1 kg or 0.1 cm, respectively. Regularly calibrated electronic scales, fixed stadiometers, and nonstretchable tapes were used according to a standard protocol. WC was measured at the midpoint between the iliac crest and the lower rib, and HC was measured at the widest circumference over the gluteal muscles. Seated blood pressure was measured after 5 minutes of rest, using a manual sphygmomanometer and suitably sized cuff. Three blood pressure measurements were taken over about a 6-minute period, with the average of the 3 measures used in analyses.

### Statistical Analyses

Six adiposity variables, either directly measured or derived, were assessed, including 2 anthropometric markers of general adiposity (height-adjusted weight [HtaW] and BMI) and 4 anthropometric markers of central adiposity (WC, HC, WHR, and WHtR). BMI was calculated as weight in kilograms divided by the square of height in meters. HtaW was calculated in men using the equation HtaW=weight in kg+0.852×(164.8−height in cm) and in women using the equation HtaW=weight in kg+0.682×(151.8−height in cm), based on the coefficients of the sex-specific linear regression models of weight on height. To limit the effects of measurement errors, very extreme or implausible values of adiposity or blood pressure were excluded (see the Table footnote for exact exclusion criteria). In addition, to limit reverse causation, those taking blood pressure–lowering medication (regularly in the last year) and those previously diagnosed with diabetes mellitus, cancer, liver cirrhosis, vascular, respiratory, or chronic kidney diseases were also excluded, as were those aged ≥90 years, or with missing data on any confounder (see below).

**Table.**
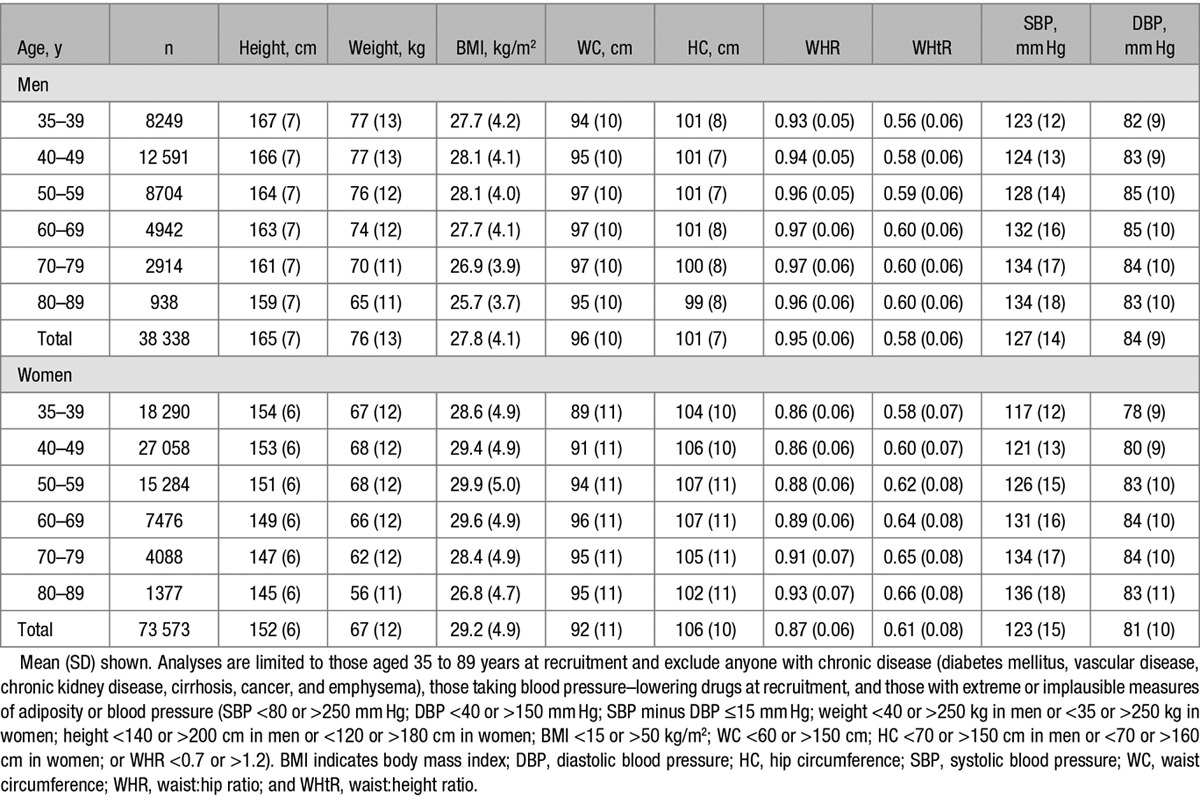
Age- and Sex-Specific Markers of Adiposity and Blood Pressure Among 111 911 Participants Who Were Aged 35 to 89 Years at Recruitment, Were Not Taking Blood Pressure–Lowering Drugs, and Had No Known Chronic Diseases

For categorical analyses of each anthropometric adiposity marker, individuals were split into 10 equally sized groups (ie, by the deciles of the sex-specific distributions). For each group, adjusted means of baseline SBP and diastolic blood pressure (DBP) were calculated using standard regression techniques, with adjustment for baseline age, residential district, education, smoking status, level of physical activity, and alcohol consumption (See Table and Table S1 in the online-only Data Supplement for groupings used for covariates). Analyses were also either adjusted for sex or done separately in men and women. Mean-adjusted blood pressure estimates together with their 95% confidence intervals were plotted against the mean adiposity levels for each group. For visual comparability of the plots between the different markers of adiposity, each horizontal axis extends across 4SDs, from −2SD below to +2SD above the mean level observed in all participants. To separate the effects of general adiposity from any independent effects of central markers of adiposity (and vice versa), analyses of each marker were done before and after further adjustment for other markers. For example, HtaW and BMI associations were estimated before and after adjustment for WHR (or waist and hip considered separately), and WC and HC associations were estimated before and after adjustment for BMI (waist and hip associations were also estimated before and after adjustment for each other). WHtR associations were adjusted for weight only (as height is already included in the derivation of WHtR). Subsequently, continuous analyses were done to estimate the mean difference in SBP and DBP associated with a 1 SD higher level of adiposity (with the same covariate adjustments as for the categorical analyses). Analyses were done using SAS version 9.3 and R version 2.11.1.

### Ethics Approval and Funding

Ethics approval was granted by the Mexican National Council of Science and Technology, the Mexican Ministry of Health, and the University of Oxford. All study participants gave informed consent. Funding was received from the British Heart Foundation, the UK Medical Research Council, The Wellcome Trust (grant 058299/Z/99), and the Mexican Ministry of Health (Secretaria de Salud, Mexico). The funders had no input on the collection, analysis, or reporting of the data.

## Results

### Study Participants

Of the 159 755 recruited participants, 4892 were excluded because they were aged ≥90 years at recruitment, had extreme measured values for blood pressure or the adiposity markers, or had missing covariate data, and a further 42 952 were excluded because they had a previous diagnosis of chronic disease or were taking blood pressure–lowering medication. The remaining 111 911 participants included 38 338 men (mean age, 51 years) and 73 573 women (mean age, 49 years).

### Baseline Markers of Adiposity, Blood Pressure, and Covariates

The age- and sex-specific mean (±SD) levels of each of the anthropometric measures, derived markers of adiposity and blood pressure, are shown in the Table. On average, across all ages, mean height was 165±7 cm in men and 152±6 cm in women, mean weight was 76±13 kg in men and 67±12 kg in women, and mean BMI was 27.8±4.1 kg/m^2^ in men and 29.2±4.9 kg/m^2^ in women. At every age, mean BMI and HC were lower, and mean WC was higher, among men, and consequently mean WHR was higher in men than in women at every age (mean across all ages, 0.95±0.06 versus 0.87±0.06 years, respectively). The WHtR increased with age in both sexes and was, on average, slightly lower among men (0.58±0.06 versus 0.61±0.08). Mean SBP increased with age, from 123±12 mm Hg at ages 35 to 39 years to 134±18 mm Hg at ages 80 to 89 years in men and, even more steeply, from 117±12 mm Hg to 136±18 mm Hg in women. DBP was less strongly, and somewhat less monotonically, related to age.

The correlations between the different adiposity markers are shown in Figures S1 and S2. In both men and women, weight, WC, and HC were all highly correlated (each pairwise age-adjusted correlation coefficient >0.80). By contrast, height was only weakly positively correlated with waist and HC in men, and nearly uncorrelated in women. BMI and WHR were weakly correlated with each other in women (adjusted *r*=0.25), but somewhat more strongly correlated in men (adjusted *r*=0.48).

The extent to which average levels of each of the adiposity markers varied depending on residential area, attained educational level, smoking status, frequency of physical activity, and alcohol consumption are shown in Tables S1 and S2. Average adiposity profiles tended to be less favorable (eg, higher general and central adiposity) among those from the poorer of the 2 studied districts (Iztapalapa), among the physically inactive and, particularly for women, among the less well educated. Anthropometric markers of adiposity were, however, largely unrelated to smoking status or alcohol consumption.

### Relevance of Adiposity to SBP

**Figure 1:**
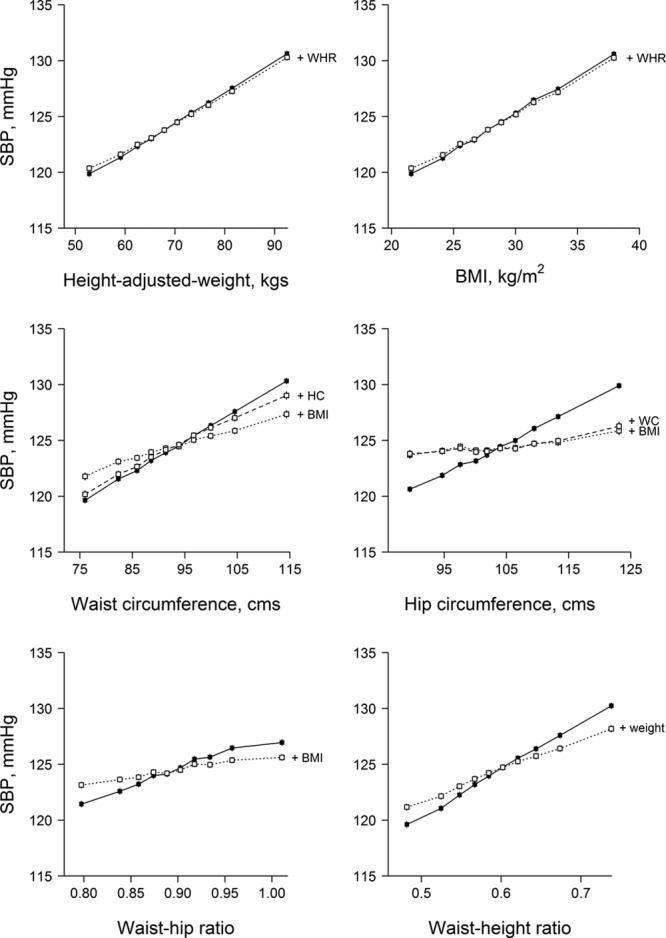
. Association between each adiposity marker and systolic blood pressure (SBP). All estimates are adjusted for age, sex, residential area, education, smoking, physical activity, and alcohol consumption. Additional further adjustments for the dotted and dashed lines are stated on the plot. Analyses exclude those taking antihypertensive treatment at recruitment, those with previous chronic disease, and those with extreme or implausible values of adiposity or blood pressure (see footnote to Table). BMI indicates body mass index; HC, hip circumference; WC, waist circumference; and WHR, waist:hip ratio.

**Figure 2:**
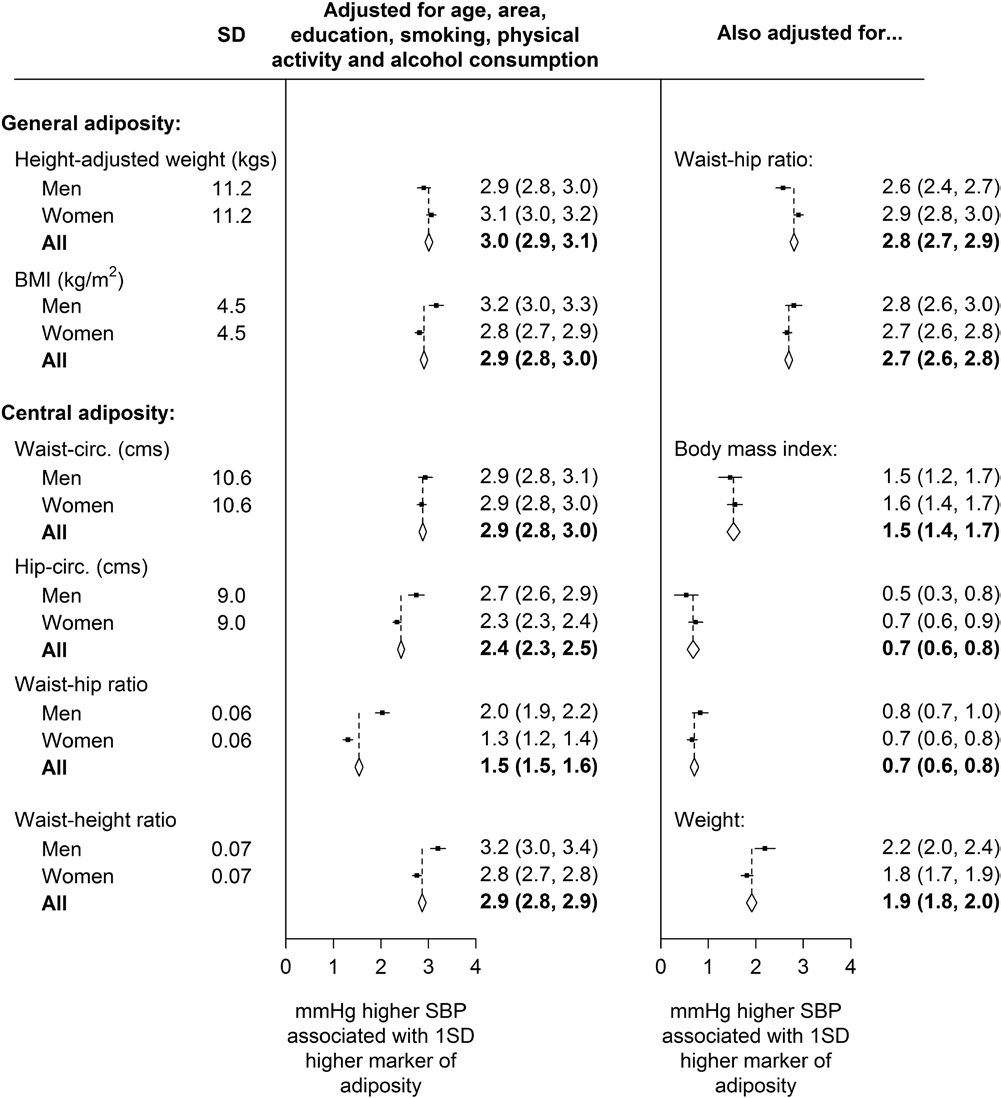
. Overall and sex-specific relevance of each adiposity marker to systolic blood pressure (SBP), before and after additional adjustment for other adiposity markers. Each diamond represents the inverse variance–weighted average of the 2 estimates above it. For each marker of adiposity, the SD shown is the average of the SD in men and the SD in women (see Table 1 for the age- and sex-specific values). Values in parentheses are 95% confidence intervals. BMI indicates body mass index.

The 2 markers of general adiposity, HtaW and BMI, were both positively, and linearly, associated with SBP, with each 1 SD higher level associated with about a 3-mm Hg higher SBP (Figures 1 and 2). Adjustment for the WHR had virtually no effect on the shape or strength of these associations (reducing the strength of the association only marginally; Figures 1 and 2).

Similarly, all 4 markers of central adiposity (WC, HC, WHR, and WHtR) were also positively, and linearly, related to SBP. However, although the strength of association for WC, HC, and WHtR was comparable with that seen for HtaW and BMI, the strength of association for the measured WHR was only about half as big (1.5 mm Hg higher SBP for each 1 SD higher WHR, with a bigger effect seen in men [2.0 mm Hg] than in women [1.3 mm Hg]; Figures 1 and 2). Moreover, adjustment for BMI (or, in the case of WHtR, for weight) reduced the magnitude of these associations considerably (Figure 2), as well as the difference between men and women in the relevance of the WHR to SBP. Among the markers of central adiposity, the association between WC and SBP was little affected by adjustment for HC, whereas the association between HC and SBP was substantially attenuated by adjustment for WC (with little further effect of adjustment for BMI; Figure 1; Figures S3 and S4). With the exception of the relevance of WHR to SBP, findings were broadly similar for men and women (Figure 2; Figures S3 and S4).

### Relevance of Adiposity to DBP

The relevance of the different anthropometric markers of adiposity to DBP is provided in Figures S5 to S8. With the exception of the WHR, each 1 SD higher adiposity level was associated with about a 2 mm Hg higher DBP (for the WHR the association was only half as strong). Again, the positive, linear associations between each marker of general adiposity and DBP were largely unaffected by adjustment for central adiposity, whereas the associations between the markers of central adiposity and DBP were greatly attenuated after adjustment for general adiposity.

### Relevance of BMI to Blood Pressure at Different Ages

The relevance of BMI to both SBP and DBP was somewhat weaker at older ages (especially among those aged ≥70 years) than at younger ages (Figure S9). Among the large numbers of participants who were aged <60 years at recruitment, however, the strength of association between BMI and blood pressure was virtually the same within each age range.

## Discussion

In this study of 110 000 Mexican adults free from known chronic diseases and not taking antihypertensive medication at the time of assessment, the anthropometric markers of adiposity that were most predictive of blood pressure were the measures of general adiposity. Within the ranges studied, there was no evidence of any threshold level below which less adiposity was not associated with lower blood pressure. On average, each 4.5 kg/m^2^ higher BMI (or each 11 kg of weight-for-height) was associated with a 3 mm Hg higher SBP and a 2 mm Hg higher DBP. For a given amount of general adiposity, any marker of central adiposity, including the ratio of waist-to-hip circumference or of waist-to-height, was much less strongly related.

Our findings in this adipose Mexican metropolitan population differ to some extent from those previously seen in other populations. For example, in a previous meta-analysis of 900 000 individuals from mostly Western populations recruited during the second half of the 20th century (and with, on average, only moderate levels of adiposity), a 5 kg/m^2^ higher BMI was associated with about a 5 mm Hg higher SBP,^1^ whereas a more contemporary study of 500 000 mostly lean Chinese adults found that 5 kg/m^2^ higher BMI was associated with an 8 mm Hg higher SBP.^13^ Our findings from this study of Mexican adults (with high levels of adiposity) give more modest estimates. Chinese dietary intake of sodium (estimated by 24-hour urinary excretion) is higher than in Mexico,^22^ as well as in many Western populations,^23,24^ and because dietary sodium predicts SBP independently of BMI,^25,26^ variation in sodium intake may partly account for these between-population differences.

Alternatively, differences may reflect the way in which blood pressure or central anthropometry was measured. By contrast with the use of automated blood pressure machines in the China Kadoorie study,^13^ blood pressure at entry into our study (as for most other cohort studies) relied on the use of manual sphygmomanometers, which are subject to observer-dependent random error, as well as digit preference.^26^ In addition, although we excluded from our analyses very extreme values, more modest measurement errors in our anthropometric measurements would tend to lead to some underestimation of the associations seen with blood pressure, with the magnitude of bias higher for those markers that are more difficult to measure precisely (eg, the WHR). Our estimates from this Hispanic population are, however, broadly comparable with estimates obtained by Mendelian-randomization studies of the relevance of adiposity to blood pressure that have been conducted in other populations. For example, a Mendelian randomization study of 14 BMI-associated single-nucleotide polymorphisms measured in 34 500 adults from 8 European population-based cohorts found that a 1 kg/m^2^ genetically elevated BMI increased SBP by 0.7 mm Hg (95% confidence intervals, 0.2–1.2).^9^ Similarly, an *FTO* polymorphism study in >145 000 participants from 30 mostly white cohorts found 1-kg/m^2^ genetically elevated BMI to be associated with a 0.9 mm Hg (95% confidence interval, 0.5–1.3) increase in SBP and 0.5 mm Hg (95% confidence interval, 0.2–0.8) increase in DBP.^10^

Our results are consistent with the hypothesis generated by other studies that, in Mexican adults, adipose tissue in general may be more relevant to blood pressure than the site of the adipose tissue per se.^27,28^ This is again consistent with analyses of 500 000 Chinese adults, in which there was little association between central markers of adiposity and blood pressure after adjustment for BMI and no suggestion of any threshold levels.^13^ However, some studies in other populations have indicated that although general and central anthropometrically defined adiposity both predict the development of hypertension,^16^ markers of central adiposity may be more discriminatory for predicting cardiovascular risk factors than markers of general adiposity.^15^

Proposed mechanisms behind the association of adiposity with blood pressure are complex and include dysfunctional adipose tissue–promoting activation of the sympathetic nervous system and the renin–angiotensin–aldosterone pathway, and adiponectin deficiency reducing nitric oxide production and increasing systemic inflammation and oxidative stress.^27,28^ We studied 6 commonly used anthropometric markers of adiposity, but others (directly measured and calculated), together with body imaging (through dual-energy X-ray absorptiometry or magnetic resonance imaging) could further disentangle the relevance to blood pressure of visceral versus other fat depots, as well as of muscle or lean mass. Such measures are, however, difficult to collect in large prospective studies in low- or middle-income countries where resources for research may be more limited. In the current study, a resurvey of 10 000 surviving participants involving additional bioimpedance measures is currently underway. Future work will therefore allow some exploration of how blood pressure in Hispanics varies with, for instance, measures of total fat mass. Elsewhere, the more advanced imaging methods of phenotyping adiposity are being used in populations with moderate levels of adiposity (such as abdominal magnetic resonance imaging currently being used in a 100 000 subset of UK Biobank participants), allowing for an even more detailed investigation of the role of adiposity on blood pressure in that mostly white population.

In our study, just 7671/111 911 participants (7%) had a BMI of <22.5 kg/m^2^, but in other populations, the relationship between BMI and blood pressure has been shown to continue down to at least 18 kg/m^2^.^1^ Despite this, however, those same studies have shown that the risk of death from cardiovascular disease is, if anything, higher at BMI <22.5 kg/m^2^ than at 22.5 kg/m^2^.^1^,^29^ Suggesting that low BMI may be correlated with other factors that have strong adverse effects on health. Adiposity influences blood lipids and glucose as well as blood pressure, and together these 3 risk factors have been estimated to explain 46% of the excess ischemic heart disease risk and 76% of the excess stroke risk associated with higher BMI (although the true percentages may be higher as these estimates do not take account of measurement errors).^30^ Cardiovascular risk reduction through weight reduction is likely to act largely through these causal pathways. Indeed, risk scores for the prediction of cardiovascular events have only tended to include markers of adiposity when information on these other risk factors is not available.^31^

## Perspectives

In Mexico, adiposity is extremely common, and its consequences through blood pressure and diabetes mellitus are a major public health concern.^3,4,17,32^ In 2013, Mexico’s National Strategy for Prevention and Control of Overweight, Obesity and Diabetes, included a range of programs related to health education, improved exercise opportunities, and taxation (eg, of sugary drinks).^33^ Our results indicate that, should this strategy be successful, it may lead to considerable population-wide benefits through blood pressure reduction as well as the benefits to be gained by reducing the incidence of diabetes mellitus.

## Acknowledgments

The chief acknowledgment is to the study participants.

## Sources of Funding

This work was supported by the Wellcome Trust (grant numbers 058299/Z/99, 090532, and 098381), the Mexican Health Ministry (SSA), the National Council of Science and Technology, Cancer Research UK, British Heart Foundation, and the UK Medical Research Council.

## Disclosures

None.

## Supplementary Material

## Novelty and Significance

### What Is New?

- A need for population-specific thresholds for central adiposity in relation to vascular risk has been proposed, but evidence from Hispanic populations is scarce.
- We examined the association of 6 markers of adiposity to blood pressure among 110 000 Mexican adults.

### What Is Relevant?

- All markers of general and central adiposity were continuously and linearly related to blood pressure, with no threshold levels below which less adiposity was not associated with lower blood pressure.
- Whereas the relevance of general adiposity to blood pressure was independent of central adiposity, much of the apparent relevance of central adiposity to blood pressure was explained by general adiposity.

### Summary

In this Mexican population, general adiposity was a stronger independent predictor of blood pressure than central adiposity in both men and women, with no evidence of any threshold effects.
