# Supplementary material for: Adiposity and Blood Pressure in 110 000 Mexican Adults
Source: Hypertension. 2017 Mar 8;69(4):608–14. doi: 10.1161/HYPERTENSIONAHA.116.08791 (PMC5344187; doi:10.1161/HYPERTENSIONAHA.116.08791)
Supplement: Supplementary file 1 [file hyp-69-608-s001.pdf]

Adiposity and blood pressure in 110,000 Mexican adults

Online Supplement

Supplementary tables

	Pg
S1. Characteristics at recruitment that might relate to the anthropometric measures among men	2
S2. Characteristics at recruitment that might relate to the anthropometric measures among women	3

Supplementary figures

S1. The anthropometric variables in relation to each other (Men)	4
S2. The anthropometric variables in relation to each other (Women)	5
S3. Association between each adiposity marker and SBP in men	6
S4. Association between each adiposity marker and SBP in women	7
S5. Association between each adiposity marker and DBP	8
S6. Overall and sex−specific relevance of each adiposity marker to DBP, before and after additional adjustment for other adiposity markers	9
S7. Association between each adiposity marker and DBP in men	10
S8. Association between each adiposity marker and DBP in women	11
S9. Relevance of body mass index to systolic and diastolic blood pressure at different ages	12

Table S1: Characteristics at recruitment that might relate to the anthropometric measures among men

	Participants	Height, cm	Weight, kg	BMI, kg/m²	Waist circ, cm	Hip circ, cm	Waist-hip ratio	Waist-height ratio
District								
Iztapalapa	21575	164 (6)	76 (11)	28.1 (4.0)	96 (10)	101 (7)	0.95 (0.05)	0.59 (0.06)
Coyoacan	16763	165 (6)	75 (11)	27.5 (4.0)	95 (10)	101 (7)	0.94 (0.06)	0.58 (0.06)
Education								
University/College	9882	167 (6)	75 (12)	27.6 (4.2)	96 (10)	102 (8)	0.94 (0.06)	0.57 (0.06)
High school	10499	165 (6)	76 (11)	27.8 (4.1)	96 (10)	101 (8)	0.95 (0.06)	0.58 (0.06)
Elementary school	14806	164 (6)	76 (11)	28.0 (4.1)	96 (10)	101 (8)	0.95 (0.06)	0.59 (0.06)
Other	3151	163 (6)	75 (12)	27.7 (4.3)	95 (10)	100 (8)	0.96 (0.06)	0.59 (0.06)
Smoking								
Never	7880	164 (6)	76 (11)	27.9 (4.1)	96 (10)	101 (8)	0.95 (0.06)	0.58 (0.06)
Former	12539	165 (6)	77 (11)	28.2 (4.1)	97 (10)	102 (7)	0.95 (0.06)	0.59 (0.06)
Current	17919	165 (6)	75 (11)	27.5 (4.1)	95 (10)	100 (8)	0.95 (0.06)	0.58 (0.06)
Regular recreational physical activity								
None	26692	165 (6)	76 (11)	28.0 (4.0)	96 (10)	101 (7)	0.95 (0.05)	0.59 (0.06)
Up to twice a week	5611	165 (6)	75 (11)	27.5 (4.1)	95 (10)	101 (7)	0.94 (0.06)	0.57 (0.06)
At least three times a week	6035	165 (6)	75 (11)	27.4 (4.1)	94 (10)	100 (8)	0.94 (0.06)	0.57 (0.06)
Alcohol consumption								
Never	2277	165 (6)	75 (11)	27.5 (4.1)	95 (10)	100 (8)	0.94 (0.06)	0.58 (0.06)
Former	3574	165 (6)	76 (11)	28.0 (4.0)	96 (10)	101 (7)	0.95 (0.05)	0.58 (0.06)
Current	32487	165 (6)	76 (11)	27.8 (4.0)	96 (10)	101 (7)	0.95 (0.05)	0.58 (0.06)

Mean (SD) shown. BMI=Body mass index. Estimates are adjusted for age and each of the other risk factors listed.

Table S2: Characteristics at recruitment that might relate to the anthropometric measures among women

	Participants	Height, cm	Weight, kg	BMI, kg/m²	Waist circ, cm	Hip circ, cm	Waist-hip ratio	Waist-height ratio
District								
Iztapalapa	45235	152 (5)	68 (11)	29.6 (4.8)	93 (11)	106 (10)	0.88 (0.06)	0.61 (0.07)
Coyoacan	28338	152 (5)	66 (11)	28.6 (4.9)	90 (11)	105 (10)	0.86 (0.06)	0.60 (0.07)
Education								
University/College	10059	155 (6)	65 (12)	27.9 (5.0)	89 (11)	104 (11)	0.85 (0.06)	0.58 (0.08)
High school	20059	153 (6)	67 (12)	28.8 (5.0)	91 (11)	105 (11)	0.86 (0.06)	0.60 (0.08)
Elementary school	34554	151 (6)	68 (11)	29.7 (4.9)	93 (11)	106 (11)	0.88 (0.06)	0.62 (0.07)
Other	8901	150 (6)	68 (12)	29.8 (5.2)	94 (12)	105 (11)	0.89 (0.06)	0.63 (0.08)
Smoking								
Never	44920	151 (5)	67 (11)	29.2 (4.9)	92 (11)	105 (11)	0.87 (0.06)	0.61 (0.07)
Former	12278	152 (5)	68 (11)	29.5 (4.8)	93 (11)	106 (10)	0.87 (0.06)	0.61 (0.07)
Current	16375	153 (6)	67 (11)	29.0 (4.9)	92 (11)	105 (11)	0.87 (0.06)	0.60 (0.07)
Regular recreational physical activity								
None	60146	152 (5)	68 (11)	29.4 (4.8)	93 (11)	106 (10)	0.87 (0.06)	0.61 (0.07)
Up to twice a week	3801	153 (5)	66 (11)	28.5 (4.8)	90 (11)	104 (10)	0.87 (0.06)	0.59 (0.07)
At least three times a week	9626	153 (6)	66 (11)	28.5 (4.9)	90 (11)	104 (11)	0.86 (0.06)	0.59 (0.07)
Alcohol consumption								
Never	18444	152 (6)	67 (11)	29.0 (4.9)	92 (11)	105 (11)	0.87 (0.06)	0.61 (0.07)
Former	3537	152 (5)	67 (11)	29.2 (4.8)	92 (11)	106 (10)	0.87 (0.06)	0.61 (0.07)
Current	51592	152 (5)	68 (11)	29.3 (4.8)	92 (11)	106 (10)	0.87 (0.06)	0.61 (0.07)

Mean (SD) shown. BMI=Body mass index. Estimates are adjusted for age and each of the other risk factors listed.

Figure S1: The anthropometric variables in relation to each other

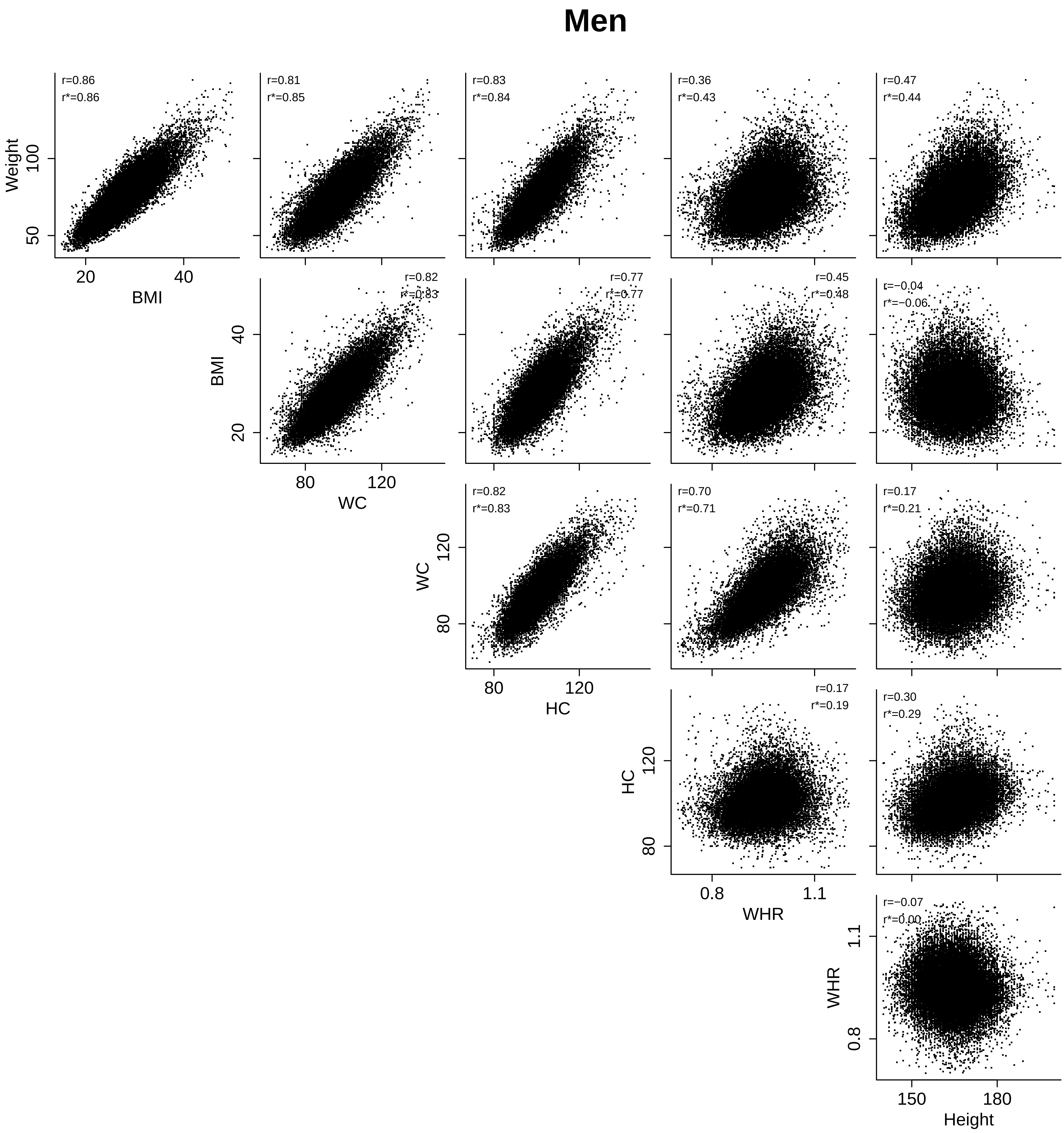

r = Pearson correlation coefficient
r* = partial (Pearson) correlation coefficent adjusted for age

Figure S2: The anthropometric variables in relation to each other

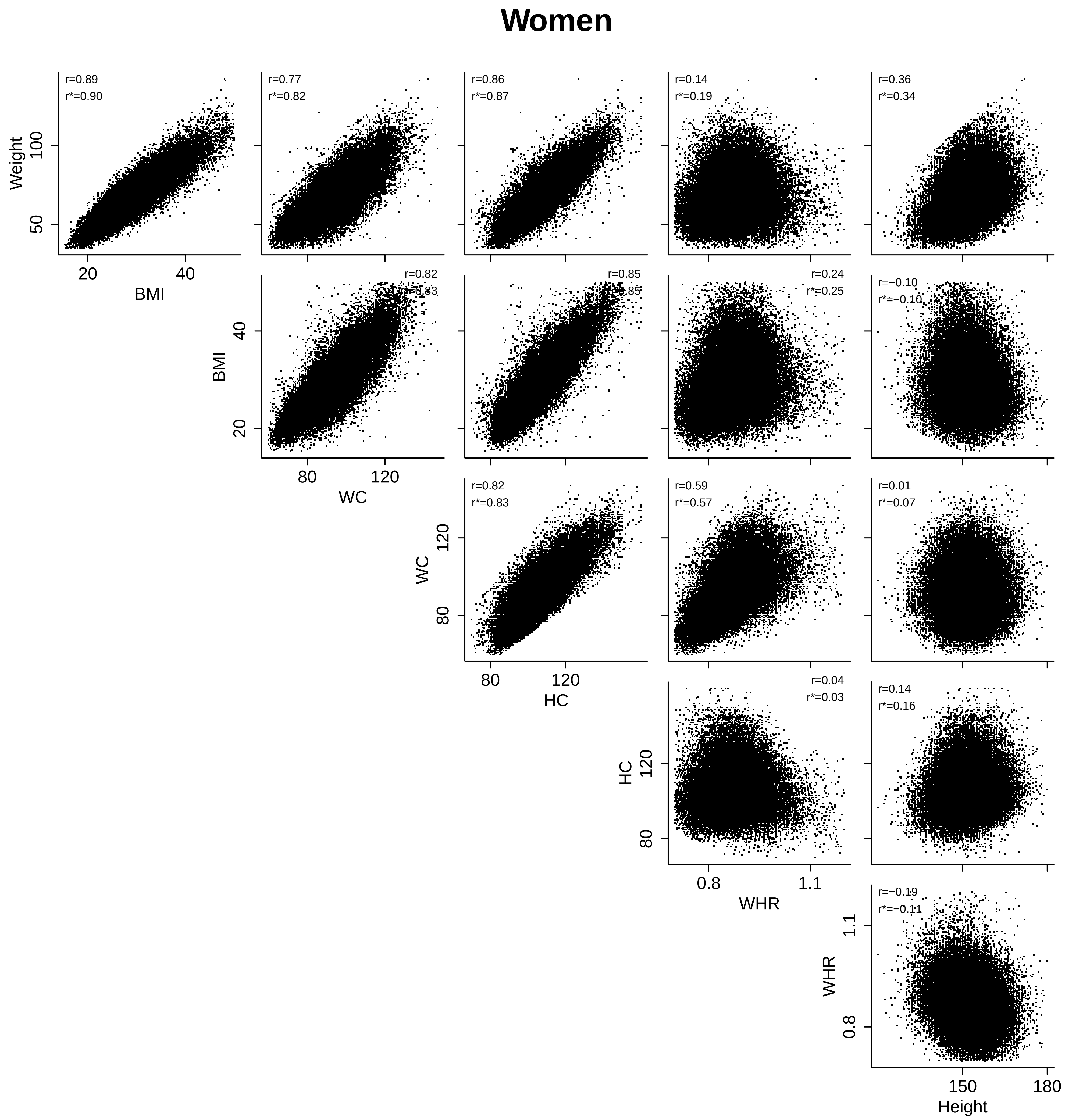

r = Pearson correlation coefficient
r* = partial (Pearson) correlation coefficent adjusted for age

Figure S3: Association between each adiposity marker and SBP in men

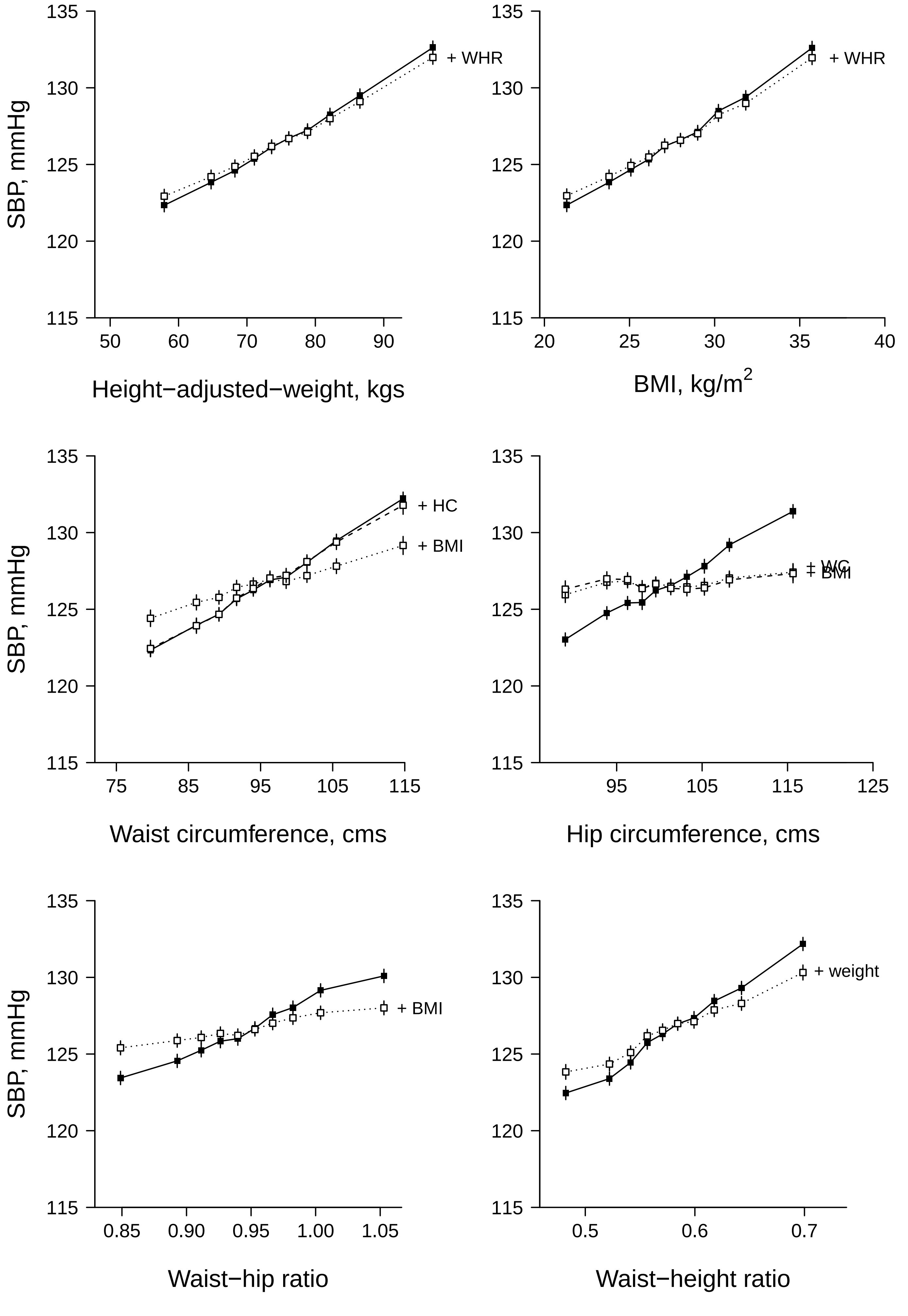

Adjustments, exclusions, axes and abbreviations as for Figure 1 in the main manuscript.

Figure S4: Association between each adiposity marker and SBP in women

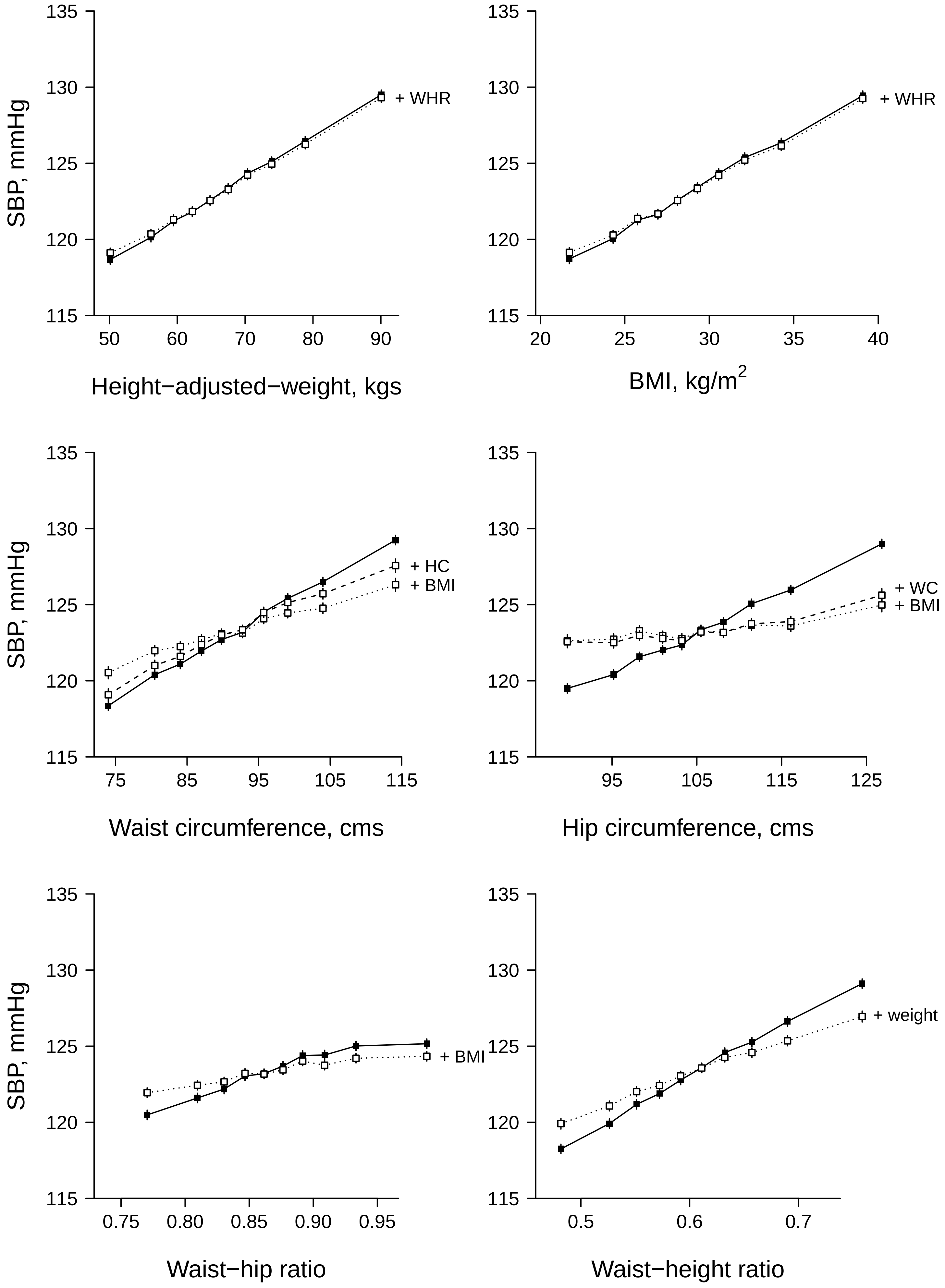

Adjustments, exclusions, axes and abbreviations as for Figure 1 in the main manuscript.

Figure S5: Association between each adiposity marker and DBP

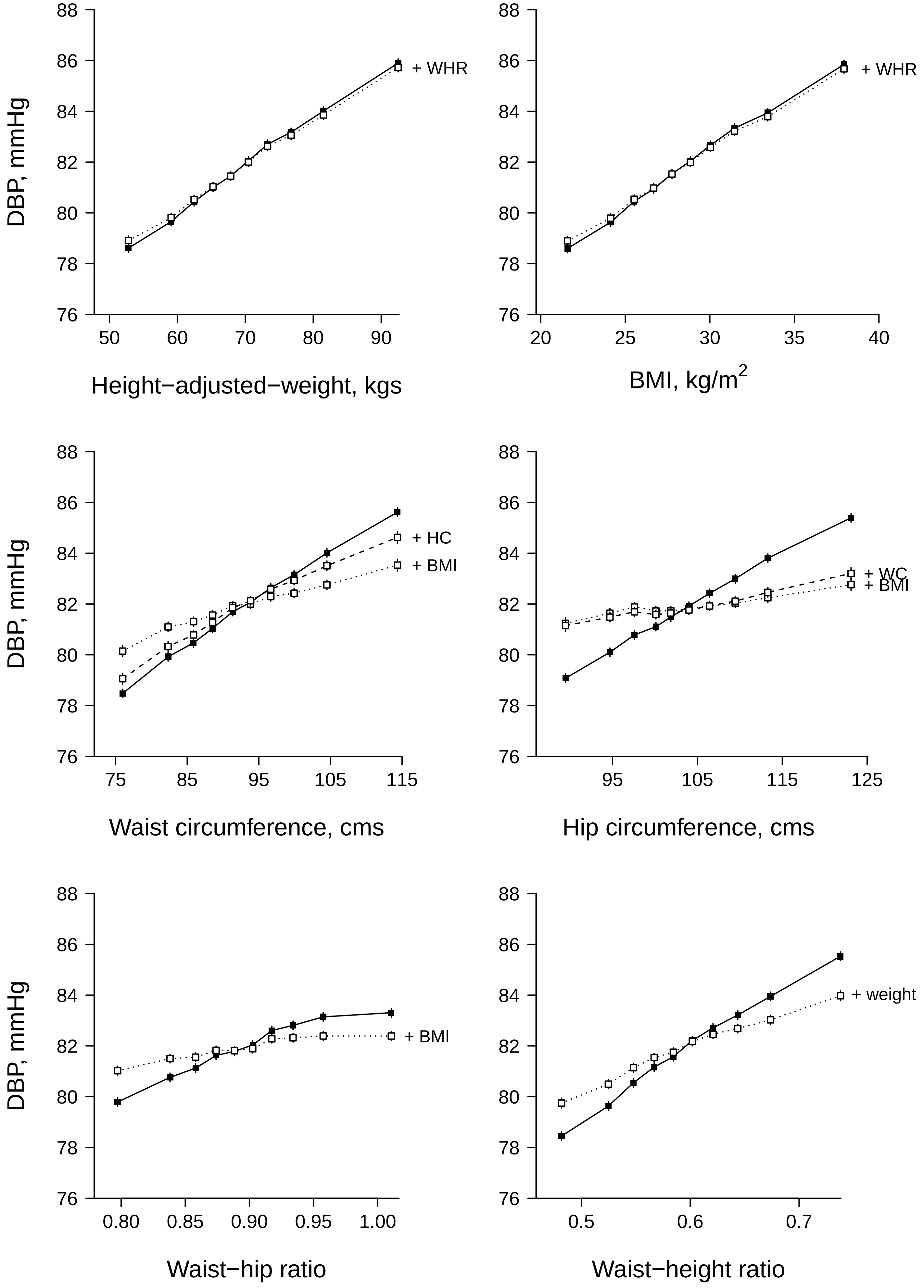

Adjustments, exclusions, axes and abbreviations as for Figure 1 in the main manuscript.

Figure S6: Overall and sex−specific relevance of each adiposity marker to DBP, before and after additional adjustment for other adiposity markers

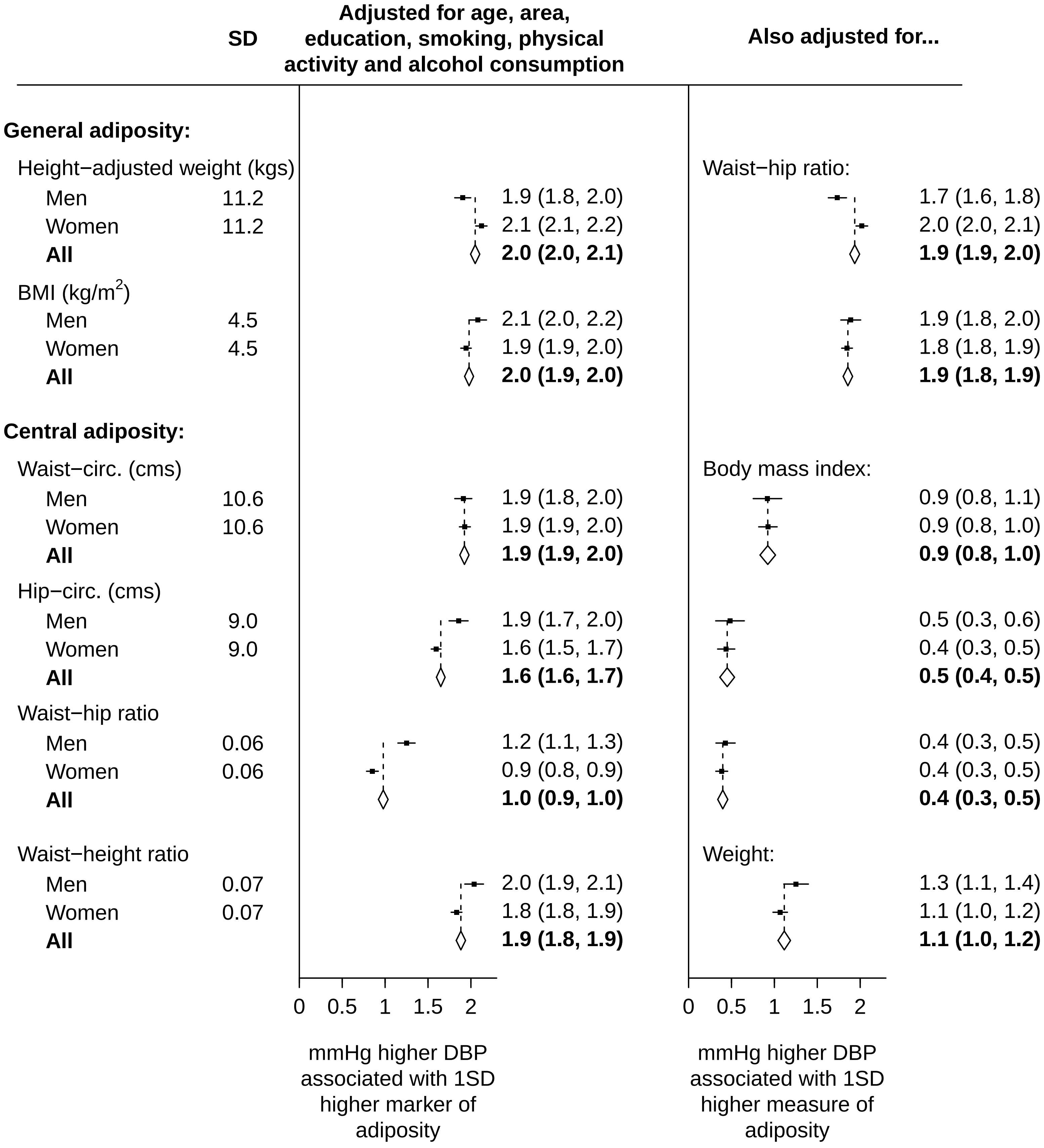

Each diamond represents the inverse−variance−weighted average of the two estimates above it.
For each marker of adiposity, the SD shown is the average of the SD in men and the SD in women (see Table 1 for the age− and sex−specific values). Values in parentheses are 95% confidence intervals.

Figure S7: Association between each adiposity marker and DBP in men

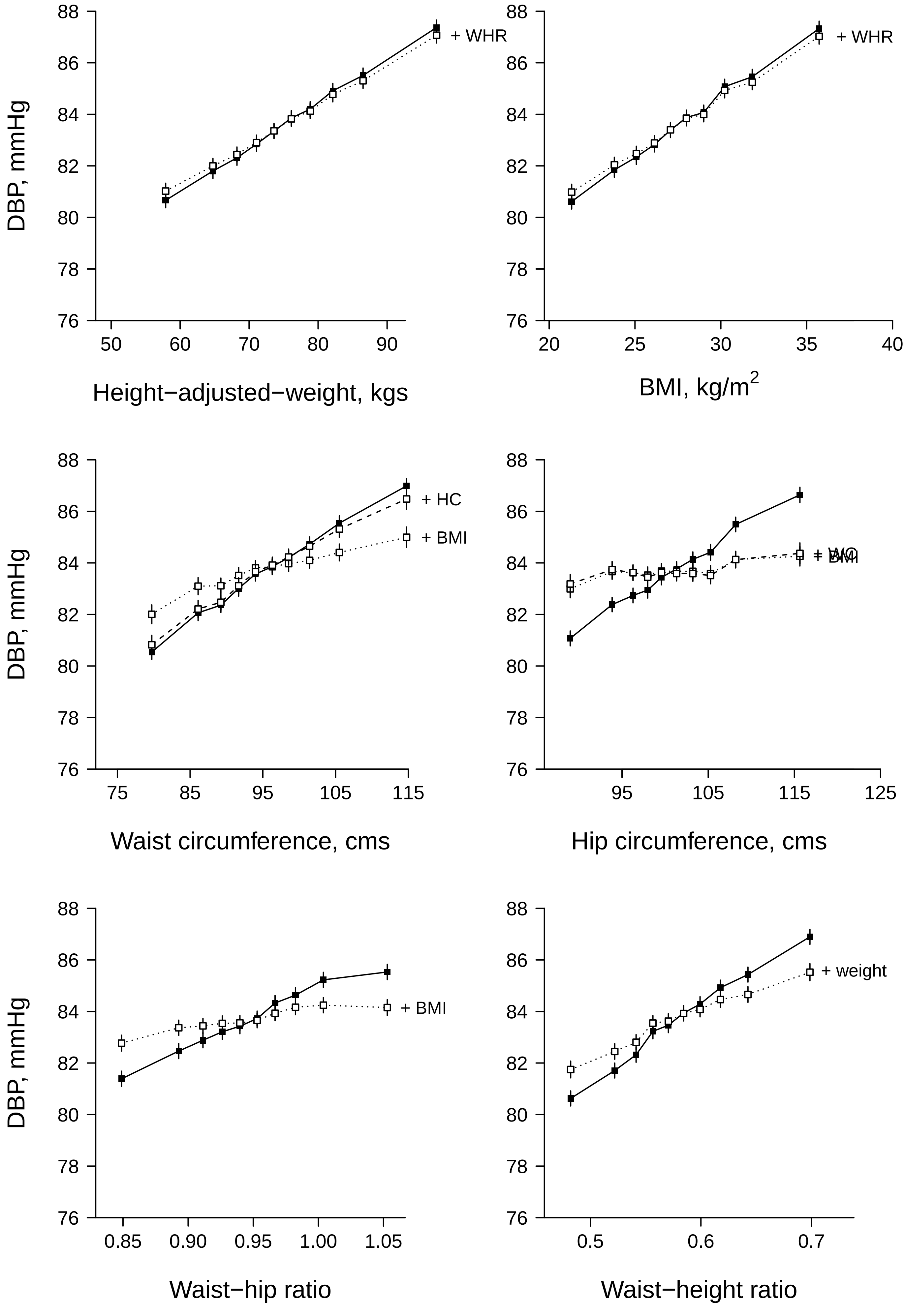

Adjustments, exclusions, axes and abbreviations as for Figure 1 in the main manuscript.

Figure S8: Association between each adiposity marker and DBP in women

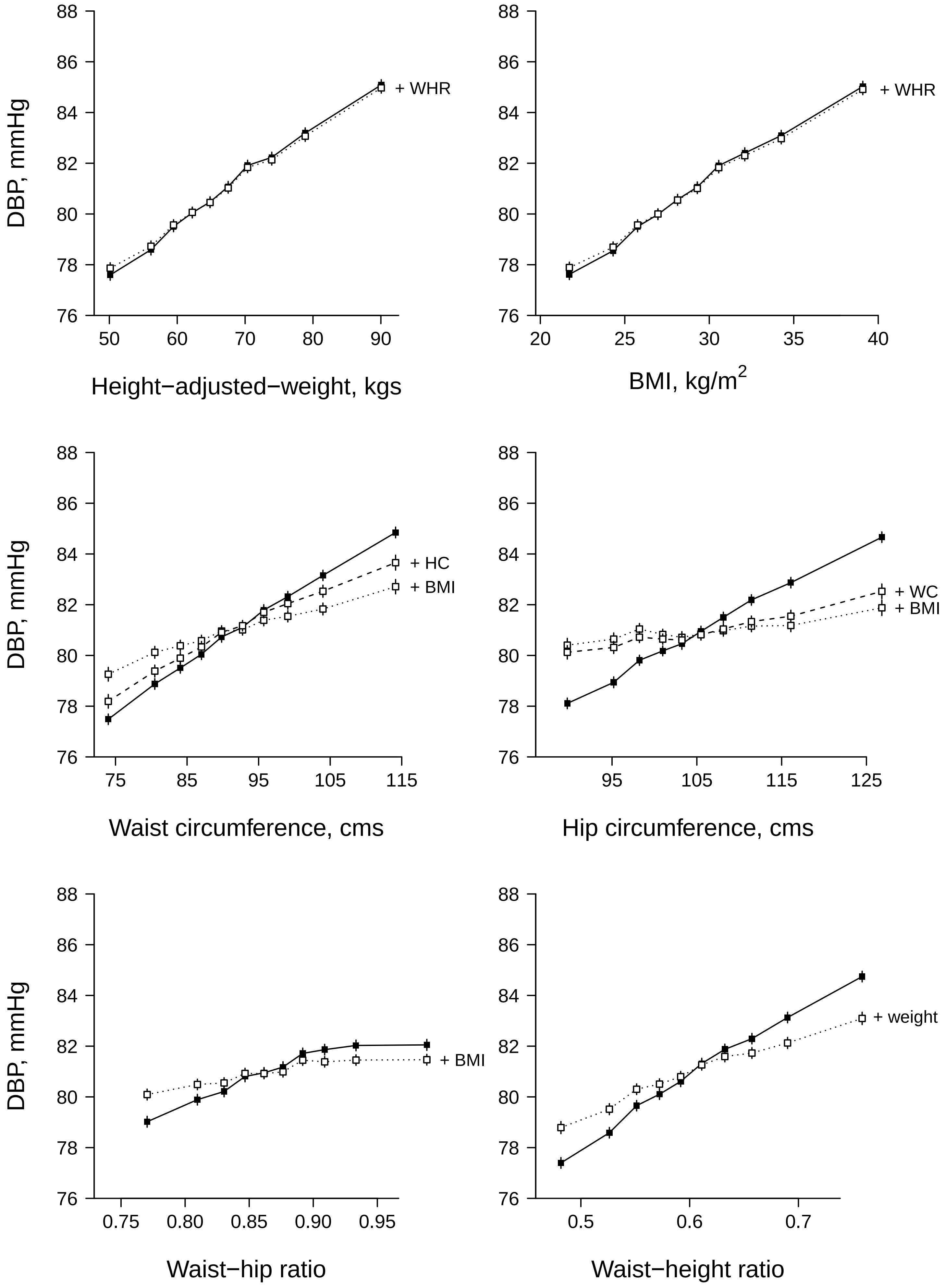

Adjustments, exclusions, axes and abbreviations as for Figure 1 in the main manuscript.

Figure S9: Relevance of body mass index to systolic and diastolic blood pressure at different ages

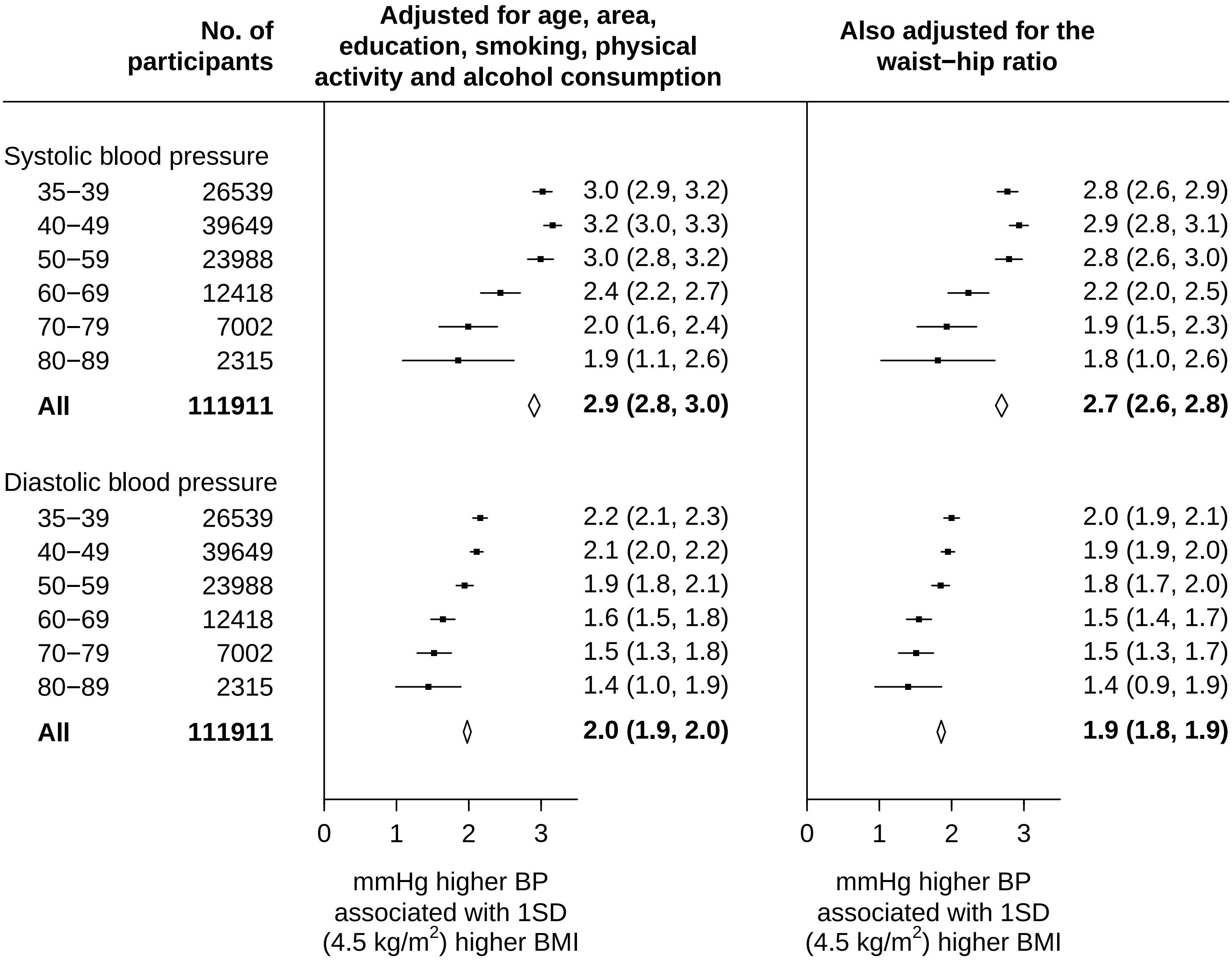

Values in parentheses are 95% confidence intervals
